# Different molecular characteristics and antimicrobial resistance profiles of *Clostridium difficile* in the Asia-Pacific region

**DOI:** 10.1080/22221751.2019.1682472

**Published:** 2019-10-30

**Authors:** Yun Luo, Elaine Cheong, Qiao Bian, Deirdre A. Collins, Julian Ye, Jeong Hwan Shin, Wing Cheong Yam, Tohru Takata, Xiaojun Song, Xianjun Wang, Mini Kamboj, Thomas Gottlieb, Jianmin Jiang, Thomas V. Riley, Yi-Wei Tang, Dazhi Jin

**Affiliations:** a Department of Microbiology, Zhejiang Provincial Center for Disease Control and Prevention, Hangzhou, People’s Republic of China; b School of Biotechnology and Biomolecular Sciences, University of New South Wales, Sydney, Australia; c Department of Infectious Diseases & Microbiology, Concord Repatriation General Hospital, Concord, Australia; d Sydney Medical School, University of Sydney, Sydney, Australia; e School of Medicine, Ningbo University, Ningbo, People’s Republic of China; f School of Medical and Health Sciences, Edith Cowan University, Joondalup, Australia; g Department of Laboratory Medicine, Busan Paik Hospital, Inje University College of Medicine, Busan, Republic of Korea; h Paik Institute for Clinical Research, Inje University College of Medicine, Busan, Republic of Korea; i Department of Microbiology, Queen Mary Hospital, Faculty of Medicine, The University of Hong Kong, Hong Kong; j Department of Infection Control, Fukuoka University Hospital, Fukuoka, Japan; k Division of Infectious Diseases, Fukuoka University School of Medicine, Fukuoka, Japan; l Centre of Laboratory Medicine, Zhejiang Provincial People Hospital, People’s Hospital of Hangzhou Medical College, Hangzhou, People’s Republic of China; m Department of Laboratory Medicine, Hangzhou First People’s Hospital, Hangzhou, People’s Republic of China; n Department of Medicine, Memorial Sloan Kettering Cancer Center, New York, NY, USA; o Department of Medicine, Weill Medical College of Cornell University, New York, NY, USA; p Key Laboratory of Vaccine, Prevention and Control of Infectious Disease of Zhejiang Province, Hangzhou, People’s Republic of China; q School of Veterinary and Life Sciences, Murdoch University, Murdoch, Australia; r Department of Microbiology, PathWest Laboratory Medicine, Nedlands, Australia; s School of Laboratory Medicine, Hangzhou Medical College, Hangzhou, People’s Republic of China

**Keywords:** *Clostridium difficile*, molecular characteristics, antimicrobial resistance, Asia-Pacific region, healthcare-associated CDI

## Abstract

Molecular epidemiology of *Clostridium difficile* infection (CDI) has been extensively studied in North America and Europe; however, limited data on CDI are available in the Asia-Pacific region. A multicentre retrospective study was conducted in this region. *C. difficile* isolates were subjected to multilocus sequence typing (ST) and antimicrobial susceptibility testing. Totally, 394 isolates were collected from Hangzhou, Hong Kong, China; Busan, South Korea; Fukuoka, Japan; Singapore; Perth, Sydney, Australia; New York, the United States. *C. difficile* isolates included 337 toxin A-positive/B-positive/binary toxin-negative (A^+^B^+^CDT^-^), 48 A^-^B^+^CDT^-^, and nine A^+^B^+^CDT^+^. Distribution of dominant STs varied geographically with ST17 in Fukuoka (18.6%), Busan (56.0%), ST2 in Sydney (20.4%), Perth (25.8%). The antimicrobial resistance patterns were significantly different among the eight sites (*χ*^2^ = 325.64, *p* < 0.001). Five major clonal complexes correlated with unique antimicrobial resistances. Healthcare-associated (HA) CDI was mainly from older patients with more frequent antimicrobial use and higher A^-^B^+^ positive rates. Higher resistance to gatifloxacin, tetracycline, and erythromycin were observed in HA-CDI patients (*χ*^2^ = 4.76-7.89, *p* = 0.005-0.029). In conclusion, multiple *C. difficile* genotypes with varied antimicrobial resistance patterns have been circulating in the Asia-Pacific region. A^-^B^+^ isolates from older patients with prior antimicrobial use were correlated with HA-CDI.

## Introduction

*Clostridium difficile* is a Gram-positive ubiquitous endospore-forming anaerobic bacterium that is a leading cause of antimicrobial-associated diarrhea and colitis [1]. *C. difficile* infection (CDI) is a toxin-mediated disease, with clinical presentations ranging from mild self-limiting diarrhea to life-threatening pseudomembranous colitis, toxic megacolon, bowel perforation and sepsis [2]. While CDI was initially mainly a healthcare-associated (HA) disease related to advanced age, exposure to antimicrobials and duration of hospitalization [3–5], there has been an increase in community-associated (CA)- CDI worldwide [6].

Recent global epidemics of CDI have shown that *C. difficile* is frequently transmitted between continents[7,8]. The epidemiology of CDI has been well documented in North America and Europe, particularly the emergence of ribotype (RT) 027 [6,9], however, the epidemiology continues to evolve. In comparison to these published in 2011 [9], data from 2012 to 2014 in Europe showed that the distribution of the most common RTs has changed and new RTs have emerged with a wide diversity of genotypes in different countries[10]. According to recent reports from the United States of America (USA) the proportion of RT 027 decreased significantly from 2011 to 2017 with increases of RTs: 106, 002, and 056 [11].

With its large population and expanding economy over recent decades, Asia has been facing challenges related to many infectious diseases, including CDI [12]. The mean overall prevalence of CDI in Asia was 14.8% with a pooled *C. difficile* incidence density of 5.3/10,000 patient days [13]. A recent prospective study in Japan found a higher *C. difficile* incidence density (7.4/10,000 patient days) than that previously reported [14]. Based on limited data from China, CDI among hospitalized patients with diarrhea had a pooled prevalence of 14.0% [15]. However, in general, there is a paucity of data on CDI in Asia owing to poor awareness among clinicians and an underestimation of contribution of CDI to antimicrobial-related diseases [12].

The molecular epidemiology of CDI in different regions in Asia is even more poorly understood than the traditional epidemiological features of the disease. There have been few studies in recent years with variable results among different countries. In China, *C. difficile* isolates with the dominant genotypes of ST2, ST3, ST35, and ST37 have been shown to be highly resistant to clindamycin and erythromycin, with high rates of multidrug resistance [15–17]. In Japan, ST17, which includes the closely related RTs smz/RT018 and smz′/QX239, was the dominant genotype, followed by RT014/ST2, RT002/ST8, and RT369/ST81 [12,14]. ST17 was also the predominant genotype in South Korea, showing high resistance to clindamycin and moxifloxacin [12,18]. However, in general, the molecular characteristics and antimicrobial resistances of *C. difficile* strains circulating in patients with CDI in the Asia-Pacific region have not been well-described, and differences between HA- and CA-CDI in the Asia-Pacific region are still unclear.

Therefore, to improve our understanding of CDI epidemiology in the Asia-Pacific region, we conducted a multicenter retrospective study at seven clinical sites and one comparative clinical site to analyze differences in molecular features and antimicrobial resistance patterns of *C. difficile* isolates and investigate risk factors associated with HA- and CA-CDI.

## Materials and methods

### *C. difficile* isolates

In total, 394 *C. difficile* isolates were collected from CDI patients on a purely random basis without selection between January 2015 and March 2016. Isolates from Perth, Australia (*n* = 31); Sydney, Australia (*n* = 54); Busan, South Korea (*n* = 50); Singapore (*n* = 29); Fukuoka, Japan (*n* = 70); and Hong Kong, China (*n* = 50) were sent to our laboratory in transport medium for anaerobes. A total of 60 isolates were from New York, USA for the purpose of comparison. Fifty isolates were cultured from fecal samples collected from CDI patients in Hangzhou, China, as previous assays reported [16]. All isolates were recovered on cefoxitin-cycloserine fructose agar plates (Oxoid, Basingstoke, United Kingdom) incubated for 48 h at 37°C in an anaerobic chamber with GENbag Anaer (bioMérieux, Marcy l’Etoile, France). Six control strains, including ATCC 43255 (RT087), ATCC BAA-1870 (ST1, RT027), and ATCC BAA-1801(RT010) were obtained from the American Type Culture Collection (ATCC; Manassas, VA, USA).

### Collection of clinical data

A standardized questionnaire was completed for each CDI patient at eight of the clinical sites, excluding Hong Kong and Singapore, to record sex, age, CDI type, CDI stage, antimicrobial treatment within the prior 8 weeks, and biochemical examinations, including white blood cells (WBCs), neutrophils, serum albumin, and creatinine, with cutoff values according to standard values [19]. Patients with diarrhea induced by other pathogens were excluded and HA- and CA-CDI were defined on the basis of the Society for Healthcare Epidemiology of America and the Infectious Diseases Society of America guidelines [3,20]. Ethical approval for *C. difficile* isolate and clinical data collection was received from the Institutional Review Boards for each respective site. This study was approved by the Ethics Committee of Zhejiang Provincial Center for Disease Control and Prevention.

### Detection of *C. difficile* toxin genes

Bacterial genomic DNA of *C. difficile* isolates and reference *C. difficile* strains (ATCC 43255, BAA-1870, and BAA-1801) were prepared using a DNeasy Blood & Tissue kit (Qiagen Inc., Valencia, CA, USA) according to the manufacturer’s protocol. The housekeeping gene *tpi*, toxin genes *tcdA* (toxin A) and *tcdB* (toxin B), and binary toxin genes *cdtA* and *cdtB* were detected by using a conventional PCR assay with the primer sequences as previously described [21,22]. The PCR amplicon sizes of *cdtA* and *cdtB* genes were 221-bp and 262-bp, respectively [21]. The *tcdA* primers amplified a 369-bp amplicon for toxin A-positive/B-positive (A^+^B^+^) strains and a 110-bp amplicon for toxin A-negative/B-positive (A^−^B^+^) strains [22]. After amplification, the PCR products were analyzed by agarose gel electrophoresis. A reference *C. difficile* strain (ATCC 43255) was used as a positive control for *tcdA* and *tcdB* and negative control for the binary toxin genes. *C. difficile* ATCC BAA-1870 was used as a positive control for the binary toxin genes, and *C. difficile* ATCC BAA-1801 was chosen as a negative control for *tcdA* and *tcdB* and the binary toxin genes. A blank, and positive and negative controls were examined in parallel for each test.

### Multilocus sequence typing (MLST)

Seven housekeeping genes (*adk*, *atpA*, *dxr*, *glyA*, *recA*, *sodA* and *tpi*) were selected, and MLST was performed as previously described [22]. PCR products were sequenced commercially by Sangon Biotech Co., Ltd. (Shanghai, China). Data for *C. difficile* alleles were deposited to determine STs in a public *C. difficile* MLST database (accessible at http://pubmlst.org/cdifficile).

### Antimicrobial susceptibility testing

Antimicrobial susceptibility testing was performed by using the agar dilution assay described by the Clinical and Laboratory Standards Institute in 2017 [23]. All isolates were subcultured on 5% Columbia blood agar (Oxoid, UK) and incubated for 48 h anaerobically at 37°C. After adjusting the turbidity to a 0.5 McFarland standard, aliquots (approximately 1 μL) of the cultures was spotted onto 1.2% Brucella agar (containing 5% lysed defiber sheep blood, 0.005‰ hemin, and 0.1% vitamin K1) plus antimicrobials at a given concentration using a multipoint inoculator and incubated anaerobically at 37°C for 48 h. The antimicrobials tested were as follows: piperacillin-tazobactam (PIP-TAZ), metronidazole, moxifloxacin, clindamycin, tetracycline, fusidic acid, vancomycin, rifampicin, erythromycin, ciprofloxacin, gatifloxacin, and levofloxacin. Interpretation of the minimal inhibitory concentration (MIC) results was based on previous studies [16]. Recommended control strains of *Bacteroides fragilis* (ATCC 25285) and *C. difficile* (ATCC 700057) were utilized. The *C. difficile* isolates resistant to three antimicrobial classes was defined as multidrug resistance (MDR) according as previously described [23].

### Data analysis

Data were analyzed using Statistical Package for Social Sciences (SPSS, Chicago, IL, USA) version 22.0. Chi-square and Fisher’s exact tests were used to analyze correlations among clinical characteristics, toxin gene profiles, MLST types and antimicrobial susceptibility profiles of *C. difficile* isolates. The t-test and nonparametric statistics were used to compare differences in ages among all sites. *P* values were calculated to assess the differences among groups, and results with *P* values of less than 0.05 were considered statistically significant.

## Results

### Analysis of clinical data at six sites

Clinical information was obtained for 315 patients with CDI at six clinical sites, excluding Hong Kong and Singapore. All clinical data were analyzed and compared among different clinical sites (Table 1). The following parameters were found to be significantly correlated with the presence of CDI among all six sites: age, CDI type, fever higher than 38.3°C, neutrophils over 70%, serum albumin less than 35 g/L, creatinine more than 111 μM, and antimicrobial use within the prior 8 weeks (Table 1). No significant differences were found in WBCs, and the proportion of WBCs (cells × 10^9^/L) of more than 10 at all sites was less than 50% (range: 26.7–44.3%). The mean age of patients with CDI was significantly younger in Hangzhou than in Busan (*t *= 5.19, *P *< 0.001), Fukuoka (*Z *= −5.18, *P *< 0.001), and New York (*Z *= −3.11, *P *< 0.001) and tended to be younger in Perth (*t *= 1.63, *P *= 0.107) and Sydney (*Z *= −1.67, *P *= 0.095). The proportions of HA-CDI were greater among older patients in Fukuoka and Hangzhou than in Busan (*χ*^2^*^ ^*= 11.33, *P *= 0.001), New York (*χ*^2^*^ ^*= 16.46, *P *< 0.001), Perth (*χ*^2^*^ ^*= 18.55, *P *< 0.001), and Sydney (*χ*^2^*^ ^*= 20.36, *P *< 0.001). The proportions of patients with CDI having a fever of higher than 38.3°C were significantly in Hangzhou and Fukuoka than in New York and Perth (*χ*^2^*^ ^*= 13.93, *P *= 0.001). Only 8.6% of patients with CDI from Fukuoka had over 70% neutrophils, which was significantly lower than those from the other five sites (*χ*^2^*^ ^*= 18.69–96.63, *P *< 0.001). The proportions of patients with CDI having serum albumin levels less than 35 g/L were much higher in Busan and Fukuoka than in the other four sites (*χ*^2^*^ ^*= 33.49–86.32, *P *< 0.001). Furthermore, only 12.0% and 11.1% of patients with CDI had a history of antimicrobial treatment within the prior 8 weeks for cases occurring in Busan and Sydney; these values were much lower than those from other sites (*χ*^2^*^ ^*= 21.61–91.67, *P *< 0.001).

**Table 1.** Clinical information of CDI patients.

Characteristics	Busan (*n* = 50)	Fukuoka (*n* = 70)	Hangzhou (*n* = 50)	New York (*n* = 60)	Perth (*n* = 31)	Sydney (*n* = 54)	Analysis results	
							*χ*^2^	*P* value
Gender, male (%)	29 (58.0%)	46 (65.7%)	32 (64.0%)	27 (45.0%)	16 (51.6%)	23 (42.6%)	10.99	0.052
Age, Mean (range)	67 (22–87)	67.1 (1–94)	53.4 (26–81)	59.6 (1–85)	58.3 (24–88)	66.2 (0.6–93)	36.96	<0.001
CDI type, HA-CDI (%)	32 (64.0%)	64 (91.4%)	40 (80.0%)	36 (60.0%)	16 (51.6%)	30 (55.6%)	31.20	<0.001
CDI stage, Primary CDI (%)	50 (100%)	70 (100%)	50 (100%)	53 (88.3%)	25 (80.6%)	50 (92.6%)	F^a^	<0.001
Fever, ≥38.3°C (%)	–	14 (20.0%)	11 (22.0%)	1 (1.7%)	2 (6.5%)	–	14.34	0.002
WBC (cells ×10^9^/L), >10 (%)	22 (44.0%)	31 (44.3%)	19 (38.0%)	16 (26.7%)	13 (41.9%)	22 (40.7%)	5.38	0.371
Neutrophils, >70% (%)	21 (42.0%)	6 (8.6%)	20 (40.0%)	57 (95.0%)	–	27 (50.0%)	98.83	<0.001
Serum albumin (g/L), <35 (%)	42 (84.0%)	62 (88.6%)	22 (44.0%)	14 (23.3%)	0	16 (29.6%)	122.51	<0.001
Creatinine (umol/L), >111 (%)	7 (14.0%)	21 (30.0%)	5 (10.0%)	0	10 (32.3%)	14 (25.9%)	29.16	<0.001
Antimicrobial used within 8 weeks, Yes (%)	6 (12.0%)	59 (84.3)	30 (60.0%)	26 (43.3%)	19 (61.3%)	6 (11.1%)	97.95	<0.001

^a^F: Fisher’s exact test.

### Antimicrobial susceptibility analysis

The antimicrobial resistance patterns of 394 *C. difficile* isolates are presented in Table S1. Antimicrobial patterns were significantly different among eight clinical sites (*χ*^2 ^= 325.64, *P *< 0.001). All isolates were susceptible to vancomycin and metronidazole, with MIC values of less than 0.5 and 2 μg/mL, respectively. No isolates were resistant to PIP-TAZ in Busan, New York, Singapore and Sydney. The rates of rifampin resistance were much lower than those of other antimicrobials in the Asia-Pacific region. The antimicrobial resistance rates were compared among three continents as follows. The rates of fusidic acid resistance in New York, Sydney, and Perth were significantly higher than those in sites from Asia (*χ*^2^*^ ^*= 125.03, *P *< 0.001). However, the rates of moxifloxacin, tetracycline, and erythromycin resistance in *C. difficile* isolates from the three above sites were much lower than those from other sites (*χ*^2^*^ ^*= 26.46–63.81, *P *< 0.001). The proportions of isolates resistant to gatifloxacin in two sites from Australia were significantly lower than those in other sites (*χ*^2^*^ ^*= 52.84, *P *< 0.001). Notably, 186 (47.2%) of these isolates were MDR, although the rate of MDR was significantly lower in isolates from three sites in the USA and Australia than those from sites in Asia (*χ*^2^*^ ^*= 51.35, *P *< 0.001).

### Genotyping analysis of *C. difficile* isolates

Of the 394 *C. difficile* isolates, 337 (85.5%) were positive for both *tcdA* and *tcdB* and negative for both *cdtA* and *cdtB* (A^+^B^+^CDT^−^), 48 (12.2%) including one from New York, four from two sites in Australia, and 43 from other sites in Asia tested negative for *tcdA* and positive for *tcdB* without *cdtA* and *cdtB* (A^−^B^+^ CDT^−^), and nine (2.3%) including two from Australia, two from sites in Asia, and five from New York were positive for *tcdA*, *tcdB*, *cdtA*, and *cdtB* (A^+^B^+^CDT^+^).

The MLST analysis identified 68 different STs, and five *C. difficile* isolates were not typed because several housekeeping genes were not amplified. ST2 (*n* = 12, 4.8%), ST3 (*n* = 15, 6.0%), ST8 (*n* = 21, 8.4%), ST17 (*n* = 42, 16.9%), ST35 (*n* = 14, 5.6%), ST37 (*n* = 20, 8.0%), ST54 (*n* = 18, 7.2%), ST63 (*n* = 9, 3.6%), and ST81 (*n* = 17, 6.8%) were the dominant genotypes in Asia. However, the different distribution of STs was found in USA and Australia as follows. ST2 (*n* = 7, 11.7%) and ST42 (*n* = 11, 18.3%) were dominant STs in New York. ST2 (*n* = 19, 22.4%) and ST8 (*n* = 10, 11.8%) were dominant genotypes in two sites in Australia. There was a significant difference on the distribution of STs among three above regions (*χ*^2^*^ ^*= 24.73, *P *< 0.001).

**Figure 1.**
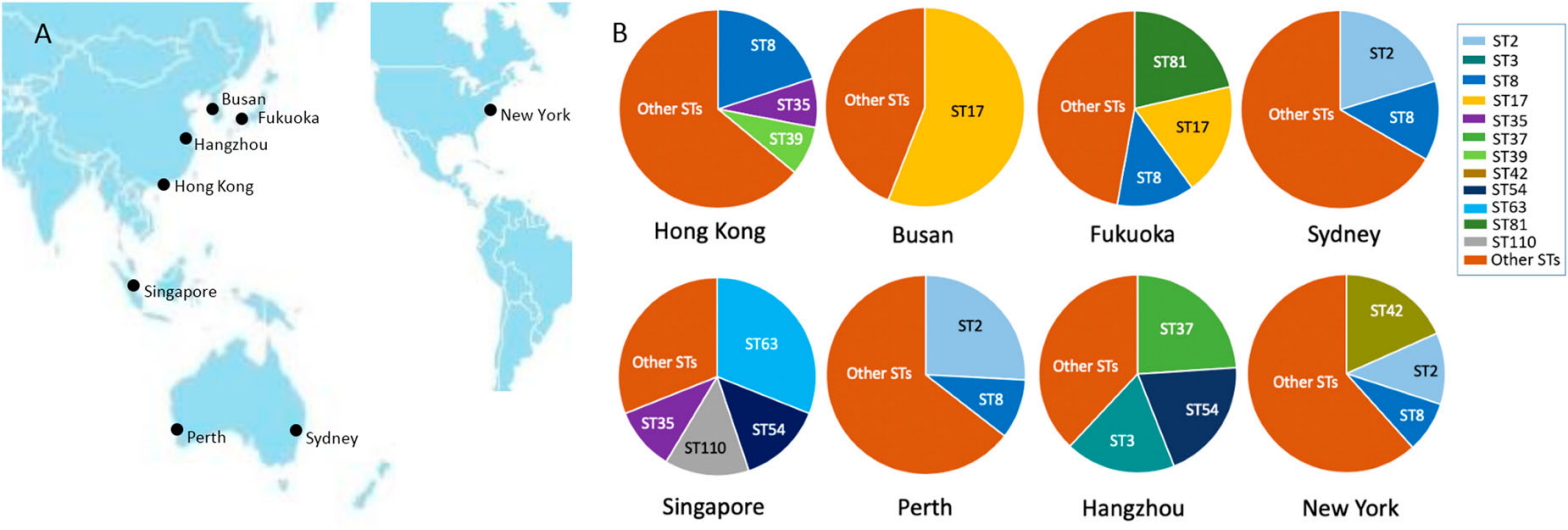
Distribution of STs at each site in this study. A: The seven sites are distributed in the Asia-Pacific region, and one comparative clinical site is New York, USA. B: Each site had various STs with different types of distribution. The major STs of *C. difficile* were labelled with different colours.

Different sites had a variety of STs, exhibiting varying distributions (Figure 1). Two isolates from New York and Sydney were ST11. The CDT-positive isolates included three ST1 isolates (one from Hong Kong, two from New York), three ST41 isolates (one from New York, two from Perth), and three other STs (ST32 and ST67 from New York, ST95 from Singapore). The dominant genotypes between sites varied significantly, with ST17 in Fukuoka (*n* = 13, 18.6%) and Busan (*n* = 28, 56.0%); ST2 in New York as above, Sydney (*n* = 11, 20.4%) and Perth (*n* = 8, 25.8%); ST8 in Hong Kong (*n* = 10, 20.0%), Fukuoka (*n* = 9, 12.8%), and Sydney (*n* = 7, 13.0%); ST63 in Singapore (*n* = 9, 31.0%); ST42 in New York as above; and ST3 (*n* = 10, 20.0%), ST37 (*n* = 13, 26.0%), and ST54 (*n* = 10, 20.0%) in Hangzhou.

### Comparison of clinical characteristics between HA- and CA-CDI

Of 315 patients with CDI with available clinical information, one patient from New York belonged to neither HA-CDI nor CA-CDI, exhibiting onset between 4 and 12 weeks after the final discharge, as previously published guidelines [3,20]. Clinical characteristics and genotypes were compared between HA- and CA-CDI for 255 patients in the Asia-Pacific region with retrievable *C. difficile* isolates (Table 2), the 59 patients from New York were also analyzed (data not shown). In comparison with CA-CDI, HA-CDI had a significantly higher frequency of age over 65 years and more frequent antimicrobial use. Moreover, patients with HA-CDI had a greater frequency of isolates with A^−^B^+^ genotype than patients with CA-CDI, although there were no significant differences in the distributions of STs between these groups (Table 2). Higher resistance rates to gatifloxacin, tetracycline, and erythromycin was observed in patients with HA-CDI (*χ*^2 ^= 4.76–7.89, *p *= 0.005–0.029). *C. difficile* isolates related to CA-CDI had much higher rates of fusidic acid resistance than isolates related to HA-CDI in the Asia-Pacific region (Table 2) and New York (data not shown). Furthermore, *C. difficile* isolates associated with CA-CDI had significantly higher rates of moxifloxacin resistance than isolates with HA-CDI in New York (Fisher’s exact test, *p *= 0.029) (data not shown).

**Table 2.** Differences of clinical characteristics, genotypes and antimicrobial resistance between HA-CDI and CA-CDI.

Characteristics	No. (%) of CDI patients		Analysis results	
	HA-CDI (*n* = 182)	CA-CDI (*n* = 73)	*χ*^2^	*P* value
Gender, male (%)	111 (61.0%)	35 (47.9%)	3.62	0.057
Age, Mean (range)	63.5 (0.8–94)	61.0 (0.6–93)		
Years of age, ≥65 (%)	87 (47.8%)	22 (30.1%)	6.64	0.010
Fever, ≥38.3°C	22 (12.1%)	5 (6.8%)	1.51	0.219
Primary CDI (%)	175 (96.2%)	66 (90.4%)	2.30	0.130
Antimicrobials used within 8 weeks (%)	93 (51.1%)	21 (28.8%)	10.51	0.001
Toxin gene pattern				
A^+^B^+^ (*n* = 216)	148 (81.3%)	68 (93.2%)	5.63	0.018
A^+^B^+^CDT^+^ (*n* = 2)	1 (0.5%)	1 (1.4%)	F^a^	0.532
A^+^B^+^CDT^−^ (*n* = 214)	147 (80.8%)	67 (91.8%)		
MLST type			F	0.119
ST2 (*n* = 27)	12 (6.6%)	15 (20.5%)	10.72	0.001
ST3 (*n* = 15)	10 (5.5%)	5 (6.8%)	0.01	0.904
ST8 (*n* = 21)	17 (9.3%)	4 (5.5%)	1.03	0.311
ST17 (*n* = 43)	32 (17.6%)	11 (15.1%)	0.23	0.628
ST35 (*n* = 12)	10 (5.5%)	2 (2.8%)	0.37	0.541
ST54 (*n* = 12)	8 (4.4%)	4 (5.5%)	<0.01	0.966
Other STs (*n* = 86)	59 (32.4%)	27 (37.0%)	0.49	0.485
A^−^B^+^(*n* = 39)	34 (18.7%)	5 (6.8%)	5.63	0.018
MLST type			F	1.000
ST37 (*n* = 19)	16 (8.8%)	3 (4.1%)	1.66	0.198
ST81 (*n* = 17)	15 (8.2%)	2 (2.7%)	1.73	0.189
Other STs (*n* = 3)	3 (1.6%)	0	F	0.560
Antimicrobial resistance rate^b^				
Fusidic acid, non-S (%)	101 (55.5%)	54 (73.9%)	5.13	0.024
Ciprofloxacin, non-S (%)	173 (95.1%)	71 (97.3%)	0.20	0.658
PIP-TAZ, non-S (%)	12 (6.6%)	4 (5.5%)	<0.01	0.963
Rifampicin, non-S (%)	11 (6.0%)	4 (5.5%)	<0.01	1.000
Moxifloxacin, non-S (%)	72 (39.6%)	20 (27.4%)	3.34	0.068
Gatifloxacin, non-S (%)	82 (45.1%)	19 (26.0%)	7.89	0.005
Clindamycin, non-S (%)	157 (86.3%)	65 (89.0%)	0.36	0.550
Levofloxacin, non-S (%)	170 (93.4%)	67 (91.8%)	0.21	0.647
Tetracycline, non-S (%)	48 (26.4%)	10 (13.7%)	4.76	0.029
Erythromycin, non-S (%)	99 (54.4%)	26 (35.6%)	7.35	0.007

^a^F: Fisher’s exact test; ^b^ S: susceptible.

### Phylogenetic analysis based on MLST

**Figure 2.**
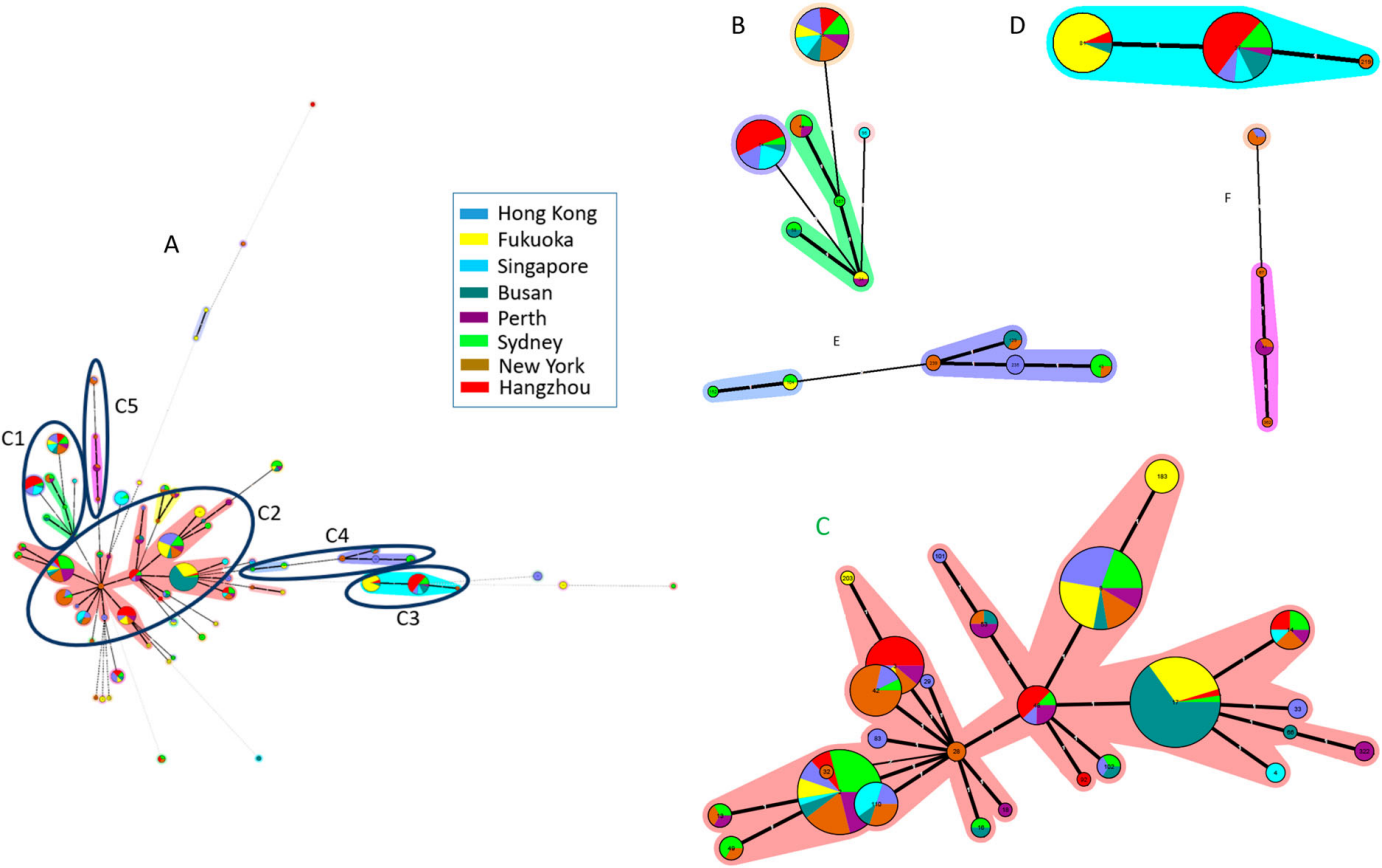
Relationship of all *C. difficile* isolates based on MLST by MST. The number in the circle shows the ST type, and the size of circle corresponds the total number of *C. difficile* isolates belonging to this ST. The number of allelic differences between STs is showed on the branches. Nodes are connected by a dashed line if the allelic difference is over two alleles. Different colours correspond to different sites. A: The MST of *C. difficile* isolates in the study, B: A part of MST-CC1 including ST34, 35, 44, 54, 58, 95, and 357; C: A part of MST-CC2 including ST2, 3, 4, 8, 13, 14, 16, 17, 18, 28, 29, 32, 33, 42, 48, 49, 53, 66, 83, 92, 101, 102, 110, 183, 203, and 322; D: A part of MST-CC3 including ST37, 81, and 219; E: A part of MST-CC4 including ST43, 104, 129, 152, 235, and 239; F: A part of MST-CC5 including ST1, 41, 67, and 362.

The genetic diversity and phylogenetic relationships of the 394 isolates were analyzed on the basis of MLST patterns. The minimum spanning tree (MST) was generated as shown in Figure 2. In total, 68 STs were distributed in the phylogenetic tree. Five major clonal complexes (CC) were artificially divided based on the discriminating ability and typeability of MLST[24,25] according the certain assay, among which more than two allelic differences existed between each of the two clusters, and there were fewer than two allelic differences between STs in the same cluster. The detailed STs were shown in each CC in Figure 2. The STs in CC1, CC2, and CC4 were distributed from various regions; all STs in CC3 belonged to A^−^B^+^ and mainly came from Hangzhou and Fukuoka (69.0%, 29/42); and STs in CC4 were predominantly from New York (62.5%, 5/8). The remaining STs were distributed outside of the five major CCs (Figure 2).

### Correlations between major genotypes and antimicrobial resistance patterns

Correlations between antimicrobial resistance and dominant genotypes are shown in Table S2. The antimicrobial patterns for A^+^B^+^ isolates were significantly different from those of A^−^B^+^ isolates (*χ*^2^*^ ^*= 35.11, *P *< 0.001). Eleven PIP-TAZ-resistant isolates were distributed in different STs, and only one isolate was found in ST37 (A^−^B^+^). All ST81 isolates were susceptible to PIP-TAZ and rifampin, but presented higher resistance rates for other antimicrobials than those of other STs. ST37 isolates possessed a higher rifampin resistance rate than other STs (Fisher’s exact test, *P *< 0.001). ST2 isolates had lower resistance rates to moxifloxacin, tetracycline, and erythromycin than other antimicrobials. ST37 and ST17 isolates showed lower fusidic acid resistance rates than other STs, except ST3 and ST54 (*χ*^2^*^ ^*= 10.35–53.25, *P *< 0.001). In general, the resistance patterns for most antimicrobials were significantly different among predominant STs (*χ*^2^*^ ^*= 53.25–109.05, *P *< 0.001).

### Correlations between major CCs and antimicrobial resistance patterns

The distributions of antimicrobial resistance patterns in five major CCs are shown in Table 3. The rates of resistance to fusidic acid, ciprofloxacin, and PIP-TAZ in CC3 were lower than those in other CCs, although there were no significant differences. However, *C. difficile* isolates had significantly higher rates of resistance to rifampin, fluoroquinolone, tetracycline, and erythromycin in CC3 in the A^−^B^+^ group than in other CCs in the A^+^B^+^ group (*χ*^2 ^= 36.84–49.29, *P *< 0.001). No isolates resistant to PIP-TAZ, metronidazole, rifampin, moxifloxacin, vancomycin, tetracycline, and erythromycin were found in CC4, in which the rate of MDR was also significantly lower than in other CCs (Fisher’s exact test, *P *= 0.001).

**Table 3.** Difference of antimicrobial resistance patterns in five clonal complexes of *C. difficile* isolates.

Antimicrobial	Clonal complexes based on MLST type (No. [%] of non-susceptible isolates)					Analysis results	
	CC1 (*n* = 52)	CC2 (*n* = 204)	CC3 (*n* = 42)	CC4 (*n* = 15)	CC5 (*n* = 8)	*χ*^2^	*P* value
Fusidic acid	30 (57.7%)	121 (59.3%)	21 (50.0%)	10 (66.7%)	6 (75.0%)	2.63	0.622
Ciprofloxacin	51 (98.1%)	199 (97.5%)	40 (95.2%)	15 (100%)	8 (100%)	F^a^	0.806
PIP-TAZ	2 (3.8%)	12 (5.9%)	1 (2.4%)	0	0	F	0.908
Metronidazole	0	0	0	0	0	N/A^b^	N/A
Rifampin	4 (7.7%)	4 (2.0%)	7 (16.7%)	0	1 (12.5%)	F	0.001
Moxifloxacin	5 (9.6%)	75 (36.8%)	26 (61.9%)	0	3 (37.5%)	36.84	<0.001
Gatifloxacin	6 (11.5%)	92 (45.1%)	31 (73.8%)	4 (26.7%)	4 (50.0%)	39.49	<0.001
Vancomycin	0	0	0	0	0	N/A^b^	N/A
Clindamycin	48 (92.3%)	186 (91.2%)	36 (85.7%)	14 (93.3%)	5 (62.5%)	F	0.115
Levofloxacin	47 (90.4%)	196 (96.1%)	39 (92.9%)	15 (100%)	8 (100%)	F	0.369
Tetracycline	23 (44.2%)	19 (9.3%)	23 (54.8%)	0	1 (12.5%)	67.92	<0.001
Erythromycin	35 (67.3%)	80 (39.2%)	35 (83.3%)	0	3 (37.5%)	49.29	<0.001
MDR	51 (98.1%)	194 (95.1%)	38 (90.5%)	10 (66.7%)	6 (75.0%)	F	0.001

^a^F: Fisher’s exact test; ^b^N/A: not applicable.

## Discussion

In recent years, an increasing number of studies has evaluated CDI epidemiology in Asia [12,26]. These have suggested that CDI is a very much under-recognized problem in Asia owing to poor clinical awareness among physicians [12]. To date, small-scale data have described CDI epidemiology within different countries in Asia. However, the molecular characteristics and antimicrobial resistances of *C. difficile* isolates in the Asia-Pacific region have not been fully elucidated, despite the large population in this region. In this retrospective study, we found that there were significant differences in ST distributions and antimicrobial resistance patterns among the seven sites in the Asia-Pacific region and New York. Patients with HA-CDI tended to be older, have more frequent antimicrobial use, exhibit lower infection rates with A^+^B^+^ genotypes, and show higher resistance to gatifloxacin, tetracycline, and erythromycin than patients with CA-CDI in Asia-Pacific region but not in New York. Five major CCs correlated with unique antimicrobial resistances, and the rifampin, fluoroquinolone, tetracycline, and erythromycin resistance rates in the A^−^B^+^ cluster were significantly higher than those in the A^+^B^+^ group.

Interestingly, a different distribution of STs was found in different regions located in separate continents including Australia, USA, and Asian countries. Moreover, specific STs were dominant in neighbouring regions. ST17 was a dominant genotype in Fukuoka and Busan, both of which were located in Northeast Asia. Sydney and Perth, two major cities in Australia, had the same dominant genotype, ST2. Similar data has been reported previously [12,18]. In addition, different sites had their own dominant genotypes, i.e. ST3, ST37, and ST54 in Hangzhou; ST63 in Singapore; ST8 in Hong Kong; and ST42 in New York. Additionally, these sites showed different antimicrobial resistance patterns.

CDI epidemiology in America has been changing in recent years. ST42 has replaced ST1 as one of the most frequent types in the USA [27], and ST42 has become one of the most common genotype in Brazil [28]. However, CDI epidemiology in China presented stable, and ST3, ST37, and ST54 have been being dominant genotypes in different regions in China [15,16]. Consequently, our study demonstrated that multiple *C. difficile* genotypes are circulating in the Asia-Pacific region and exhibit unique molecular characteristics at each site. *C. difficile* isolates with the same STs may be transmitted between different regions through food trade and travel in the Asia-Pacific region as previously reported [26]. Further studies focusing on analysis of whole-genome sequences combined with epidemiological data are required to investigate genetic relationship between different sites for the same STs.

There were statistically significant differences in antimicrobial resistance patterns not only among different sites but also among the major STs identified in our study. Our previous study also showed that the major genotypes were associated with significantly different antimicrobial resistance patterns in comparison with other genotypes in *C. difficile* in Eastern China [16]. Antimicrobial resistance mechanisms in *C. difficile* have been investigated and associated with genes and gene mutations [29]. Different STs obtained various mobile genetic elements containing different antimicrobial resistance-related genes at the different time in the course of genome evolution, maybe resulting in significant differences in antimicrobial resistance patterns among the STs [8,30]. Although extremely high rates of resistance to clindamycin, and levofloxacin were observed at all sites, New York, Sydney, and Perth showed lower rates for moxifloxacin, tetracycline, and erythromycin resistance than isolates from Asian locations. The low antimicrobial resistance rates in these sites could have several explanations. In Australia, the use of fluoroquinolones has historically been regulated and restricted in both humans and food-producing animals [31]. Access to antimicrobials for clinical use can only be obtained through physician prescription and antimicrobials are not available for over the counter purchase. Furthermore, antimicrobial stewardship programmes were established during the early stages of epidemic outbreaks and strictly implemented in the USA and Australia, respectively [32–35]. As inappropriate use of antimicrobials has been a major concern in recent years in Asia, a stewardship approach to antimicrobial use in humans and animals, when combined with enhanced infection control, may help to decrease antimicrobial resistance rates in the future.

Even though some clinical parameters were found to be significantly correlated with CDI, small numbers of samples might lead to possible bias in our data analysis results between sites. Thus, more clinical samples should be collected to investigate differences on clinical parameters of CDI patients among different countries in the Asia-Pacific region. Notably, we found that the mean age of patients with CDI in Hangzhou was younger than that in other sites despite small numbers of samples involved in this study. Similar results have been reported in the other studies of CDI epidemiology in China [16,36,37]. Interestingly, we also found the high rates of HA-CDI with over 50% in each site, and HA-CDI tended to be older patients with more frequent antimicrobial use. Furthermore, *C. difficile* A^−^B^+^ genotypes infected more patients with HA-CDI, with higher rates of gatifloxacin, tetracycline, and erythromycin resistance, than patients with CA-CDI in the Asia-Pacific region. These above molecular characteristics of CDI were obviously different from those in New York. To the best of our knowledge, this was the first study elucidating the differences between patients with HA- and CA-CDI in the Asia-Pacific region and differences on molecular characteristics of CDI in between the Asia-Pacific region and the other region. While healthcare associated acquisition has been the primary pathway for CDI increasing incidence densities in the USA [38], recent data indicated that over 40% of CDIs in the USA were community-associated [6,39]; in Europe, 76.4% of CDI cases were healthcare-associated [40]. However, the main transmission pathway of CDI is still unknown in Asia, particularly China. Large-scale CDI epidemiological investigations are required to clarify the main route of transmission for CDI.

Our study had several limitations. First, all *C. difficile* isolates used in this study were not collected at the same period. CDI epidemiology is dynamically changing; thus, the distributions of genotypes and antimicrobial resistance patterns may have shown slight bias during data analysis. Second, this was a retrospective study involving relatively small numbers of cases recruited from each site. There were no detailed set inclusion or exclusion criteria thereby potentially resulting in a possible bias in our data analysis results between sites. Third, as there is a paucity of data on clinical outcomes in CDI patients, the differences of clinical outcomes among different regions and among CDIs with different molecular genotypes were not analyzed. Fourth, our study lacked some sites in Asian countries and did not cover the whole Asia-Pacific region. Thus, further investigations are required to clarify the differences of clinical outcomes induced by various genotypes of *C. difficile* in different regions, and confirm the molecular characteristics of *C. difficile* isolates in the Asia-Pacific region.

In conclusion, this was the first multicenter retrospective study in the Asia-Pacific region evaluating differences in the molecular features and antimicrobial resistance patterns of *C. difficile* isolates and investigating risk factors in patients with HA- and CA-CDI. Multiple *C. difficile* genotypes with varied antimicrobial resistance patterns have been circulating in the Asia-Pacific region. A^−^B^+^ isolates in older people and individuals with prior antimicrobial use were associated with HA-CDI. Consequently, CDI has a major presence in the Asia-Pacific region, and it is necessary to initiate CDI surveillance through international collaboration and implement antimicrobial stewardship programmes in order to prevent and control CDI.

## Supplementary Material

Supplemental Material
