# Supplementary material for: Different molecular characteristics and antimicrobial resistance profiles of Clostridium difficile in the Asia-Pacific region
Source: Emerg Microbes Infect. 2019 Oct 30;8(1):1553–62. doi: 10.1080/22221751.2019.1682472 (PMC6830245; doi:10.1080/22221751.2019.1682472)
Supplement: Supplemental Material [file TEMI_A_1682472_SM0950.docx]

Table S1. The antimicrobial resistance patterns of 394 *C.difficile* isolates at eight sites

| Antimicrobial |  | No. (%) of non-susceptible isolates | | | | | | | | Analysis results | |
| --- | --- | --- | --- | --- | --- | --- | --- | --- | --- | --- | --- |
|  | Total no. (%) of isolates | Busan  South Korea | Fukuoka  Japan | Hangzhou  China | Hong Kong  China | New York  USA | Perth  Australia | Singapore  Singapore | Sydney  Australia | *χ*^2^ | *P* value |
|  | (n=394) | (n=50) | (n=70) | (n=50) | (n=50) | (n=60) | (n=31) | (n=29) | (n=54) |  |  |
| Fusidic acid | 235 (59.6%) | 7 (14%) | 52 (74.3%) | 10 (20%) | 5 (10%) | 58 (96.7%) | 30 (96.8%) | 22 (75.9%) | 51 (94.4%) | 215.62 | <0.001 |
| Ciprofloxacin | 383 (97.2%) | 50 (100%) | 69 (98.6%) | 42 (84%) | 50 (100%) | 60 (100%) | 31 (100%) | 29 (100%) | 52 (96.3%) | F^a^ | <0.001 |
| PIP-TAZ | 17 (4.31%) | 0 | 7 (10%) | 1 (2%) | 1 (2%) | 0 | 8 (25.8%) | 0 | 0 | F | <0.001 |
| Metronidazole | 0 | 0 | 0 | 0 | 0 | 0 | 0 | 0 | 0 | N/A^b^ | N/A |
| Rifampin | 19 (4.8%) | 6 (12%) | 2 (2.9%) | 5 (10%) | 3 (6%) | 0 | 0 | 1 (3.5%) | 2 (3.7%) | F | 0.040 |
| Moxifloxacin | 127 (32.2%) | 33 (66%) | 45 (64.3%) | 9 (18%) | 19 (38%) | 6 (10%) | 3 (9.7%) | 10 (34.5%) | 2 (3.7%) | 105.41 | <0.001 |
| Gatifloxacin | 158 (40.1%) | 35 (70%) | 47 (67.1%) | 14 (28%) | 23 (46%) | 24 (40%) | 3 (9.7%) | 10 (34.5%) | 2 (3.7%) | 85.80 | <0.001 |
| Vancomycin | 0 | 0 | 0 | 0 | 0 | 0 | 0 | 0 | 0 | N/A | N/A |
| Clindamycin | 351 (89.1%) | 46 (92%) | 66 (94.3%) | 33 (66%) | 47 (94%) | 55 (91.7%) | 26 (83.9%) | 27 (93.1%) | 51 (94.5%) | 34.39 | <0.001 |
| Levofloxacin | 370 (93.9%) | 50 (100%) | 69 (98.6%) | 38 (76%) | 50 (100%) | 54 (90%) | 31 (100%) | 29 (100%) | 49 (90.7%) | F | <0.001 |
| Tetracycline | 74 (18.8%) | 6 (12%) | 23 (32.9%) | 22 (44%) | 11 (22%) | 1 (1.7%) | 6 (19.4%) | 4 (13.8%) | 1 (1.9%) | 53.93 | <0.001 |
| Erythromycin | 186 (47.2%) | 33 (66%) | 43 (61.4%) | 31 (62%) | 27 (54%) | 17 (28.3%) | 6 (19.4%) | 17 (58.6%) | 12 (22.2%) | 51.35 | <0.001 |

^a^ F: Fisher’s exact test; ^b^ N/A: not applicable

Table S2. Correlations between major MLST types and antimicrobial resistance patterns of *C. difficile* isolates

| Antimicrobial |  |  | | MLST type (No. [%] of non-susceptible isolates) | | | | | | | | | | | | | | Analysis results | |
| --- | --- | --- | --- | --- | --- | --- | --- | --- | --- | --- | --- | --- | --- | --- | --- | --- | --- | --- | --- |
|  | A^+^B^+^ | | | | | | | | A | |  | |  | | A^-^B^+^ | | |  |  |
|  | ST2  (n=38) | | ST3  (n=19) | | ST8  (n=36) | ST17  (n=43) | ST35  (n=23) | ST42  (n=14) | | ST54  (n=19) | |  | | ST37  (n=24) | | ST81  (n=17) | *χ*^2^ | | *P* value |
| Fusidic acid | 28 (73.7%) | | 8 (42.1%) | | 24 (66.7%) | 9 (20.9%) | 13 (56.5%) | 12 (85.7%) | | 8 (42.1%) | |  | | 5 (20.8%) | | 15 (88.2%) | 53.25 | | <0.001 |
| Ciprofloxacin | 38 (100%) | | 18 (94.7%) | | 36 (100%) | 43 (100%) | 22 (95.7%) | 12 (85.7%) | | 19 (100%) | |  | | 22 (91.6%) | | 17(100%) | F^a^ | | 0.016 |
| PIP-TAZ | 2 (5.3%) | | 0 | | 2 (5.6%) | 4 (9.3%) | 2 (8.7%) | 0 | | 0 | |  | | 1 (4.2%) | | 0 | F | | 0.815 |
| Metronidazole | 0 | | 0 | | 0 | 0 | 0 | 0 | | 0 | |  | | 0 | | 0 | N/A^b^ | | N/A |
| Rifampin | 1 (2.6%) | | 0 | | 0 | 1 (2.3%) | 2 (8.7%) | 0 | | 2 (10.5%) | |  | | 7 (29.2%) | | 0 | F | | <0.001 |
| Moxifloxacin | 2 (5.3%) | | 4 (21.1%) | | 19 (52.8%) | 37 (86%) | 4 (17.4%) | 2 (14.3%) | | 0 | |  | | 9 (37.5%) | | 17(100%) | 109.05 | | <0.001 |
| Gatifloxacin | 6 (15.8%) | | 5 (26.3%) | | 22 (61.1%) | 39 (90.7%) | 5 (21.7%) | 7 (50.0%) | | 1 (5.3%) | |  | | 13 (54.2%) | | 17(100%) | 92.02 | | <0.001 |
| Vancomycin | 0 | | 0 | | 0 | 0 | 0 | 0 | | 0 | |  | | 0 | | 0 | N/A^b^ | | N/A |
| Clindamycin | 34 (89.5%) | | 17 (89.5%) | | 33 (91.7%) | 42 (97.7%) | 21 (91.3%) | 14 (100%) | | 17 (89.5%) | |  | | 18 (75.0%) | | 17(100%) | F | | 0.116 |
| Levofloxacin | 37 (97.4%) | | 17 (89.5%) | | 36 (100%) | 43 (100%) | 21 (91.3%) | 14 (100%) | | 17 (89.5%) | |  | | 21 (87.5%) | | 17(100%) | F | | 0.032 |
| Tetracycline | 2 (5.3%) | | 3 (15.8%) | | 2 (5.6%) | 6 (14%) | 14 (60.9%) | 0 | | 9 (47.4%) | |  | | 11 (45.8%) | | 12 (70.6%) | 68.14 | | <0.001 |
| Erythromycin | 2 (5.3%) | | 14 (73.7%) | | 10 (27.8%) | 38 (88.4%) | 17 (73.9%) | 3 (21.4%) | | 17 (89.5%) | |  | | 17 (70.8%) | | 17(100%) | 106.78 | | <0.001 |

^a^ F: Fisher’s exact test; ^b^ N/A: not applicable
